# Necrology

**Published:** 1891-06

**Authors:** 


### NECROLOGY.

### JOSEPH LEIDY.

Joseph Leidy, M.D., L.L.D., died on April 30, of an affection of the kidneys, followed by congestion of the brain. Dr. Leidy was born in Philadelphia, September 9, 1823. His ancestors on both sides, were Germans from the Valley of the Rhine. At the age of sixteen, he left school intending to become an artist. Employed in a drug store, for the next few years, he became interested in botany and minerals and commenced the study of medicine in 1840. His graduating thesis was on the Comparative Anatomy of the Eye of Vertebrated Animals. Immediately upon graduation he was appointed an assistant in the laboratories of the Medical Department of the University of Pennsylvania and, shortly afterwards, dropping the practice of medicine, he devoted himself entirely to study and teaching until his death. From his universal discoveries and knowledge of Natural History, his work in Paleontology is especially interesting to the veterinarian.

In September, 1847, he published in the proceedings of the Academy of Natural Sciences his first Paleontological paper, entitled "On the Fossil Horse of America." The existence of remains of extinct horses on the American continent had been regarded with incredulity in consequence of the entire disappearance of these animals in after ages. The paper consists of descriptions and figures of specimens contained in the museum of the Academy of Natural Sciences, of Philadelphia, some of which the author regarded as belonging to the South American form described by Owen and others as indicating a new species, for which he proposed the name Equus Americanus.

Long before the active exploration of the West had added so immensely to the knowledge of the extinct fauna of that region, he had determined the former existence in a tropical climate on the western slope, of the lion, tiger, camel, the horse, rhinocerous, and many other forms having no immediate existing representatives. In 1853 the Smithsonian Institution published his memoranda on the extinct species of the American ox, and in the following year the elaborate Ancient Fauna of Nebraska.

"The Extinct Mammalian Fauna of Dakota and Nebraska," appeared as the seventh volume of the journal of the Academy. The work, a quarto of 472 pages, illustrated by 30 lithographic plates, was the result of the gradual accumulation of material during twenty-three years. This elaborate work was followed in 1873 by one of equal importance, under the title, "Contributions to the Extinct Vertebrate Fauna of the Western Territories." It forms the first volume of the superb quarto reports of the survey of the Territories under Dr. Hayden, and consists of 354 pages and 37 plates. For many years after the publication of his paper on "Fossil Horses," in 1847, Dr. Leidy was almost the only American author whose attention was given to the study of the extinct vertebrata.

His memoir entitled "Fresh Water Rhizopods of North America," is considered one of the best illustrations of Dr. Leidy's qualities as a naturalist, and a most enduring monument to his industry.

At the organization of the Veterinary Department of the University of Pennsylvania, Dr. Leidy, having a great interest in its plans, became a member of the Faculty, as Professor of

Zoology, and hoped, with the Dean at that time, for a museum to combine the collection of Comparative Anatomy and those of Veterinary School. As a teacher he was admired and loved by all students; as a man he was honored by all who knew him; as a scientist he was an example of methodical work, minute in details but ever ready to lend aid to those around him, and he was more than human in his integrity, honesty, and freedom from the petty jealousies of the pseudo-political world which darkens public institutions and agregations of selfish men.

An autopsy was held in accordance with the rule of the American Neurological Society, of which the deceased was President and which society designs to examine for comparative purposes the brains of prominent men. By his own instructions his body was then cremated. R. S. H,

---

### Leon Lafosse.

*Former Professor and Director of the Veterinary School at Toulouse, France.*

Prof. Lafosse died on March 18, 1891; he was born in the department of Aisne, France, in 1816. A celebrated practitioner, teacher and writer in veterinary medicine for over a half century, Prof. Lafosse's name will stand high in the history of veterinary medicine, not only in his own country, but throughout the whole world. He is the author of 112 publications, on subjects of Surgery, Veterinary Pathology, and Breeding, of which among others perhaps, the most important subjects, were those of Vaccinia, Tuberculosis, Cattle breeding and Phylloxera.

---

### E. Nostrand, D. V. S.

After a practice of over fifty years in the city of New York, Doctor Elbert Nostrand, died of pneumonia on April 14, 1891, at the advanced age of eighty-three years and one month. Born in Springfield, Long Island, he, at an early age, began practice in New York city, and was one of the first American veterinary graduates, receiving his diploma in 1865 from the New York College of Veterinary Surgeons. Dr. Nostrand even at his advanced age, in fact, to within a few days of his sickness, was still in active practice, and many New Yorkers often met him but a short time before his death, in the performance of his professional duties.

